# Convolutional neural networks for automatic image quality control and EARL compliance of PET images

**DOI:** 10.1186/s40658-022-00468-w

**Published:** 2022-08-09

**Authors:** Elisabeth Pfaehler, Daniela Euba, Andreas Rinscheid, Otto S. Hoekstra, Josee Zijlstra, Joyce van Sluis, Adrienne H. Brouwers, Constantin Lapa, Ronald Boellaard

**Affiliations:** 1 Nuclear Medicine, Medical Faculty, University of Augsburg, Augsburg, Germany; 2 Medical Physics and Radiation Protection, University Hospital Augsburg, Augsburg, Germany; 3 Department of Radiology and Nuclear Medicine, VU University Medical Center, Amsterdam, The Netherlands; 4 Department of Nuclear Medicine and Molecular Imaging, Medical Imaging Center, University Medical Center Groningen, University of Groningen, Groningen, The Netherlands

**Keywords:** PET, Image quality, Convolutional neural network

## Abstract

**Background:**

Machine learning studies require a large number of images often obtained on different PET scanners. When merging these images, the use of harmonized images following EARL-standards is essential. However, when including retrospective images, EARL accreditation might not have been in place. The aim of this study was to develop a convolutional neural network (CNN) that can identify retrospectively if an image is EARL compliant and if it is meeting older or newer EARL-standards.

**Materials and methods:**

96 PET images acquired on three PET/CT systems were included in the study. All images were reconstructed with the locally clinically preferred, EARL1, and EARL2 compliant reconstruction protocols. After image pre-processing, one CNN was trained to separate clinical and EARL compliant reconstructions. A second CNN was optimized to identify EARL1 and EARL2 compliant images. The accuracy of both CNNs was assessed using fivefold cross-validation. The CNNs were validated on 24 images acquired on a PET scanner not included in the training data. To assess the impact of image noise on the CNN decision, the 24 images were reconstructed with different scan durations.

**Results:**

In the cross-validation, the first CNN classified all images correctly. When identifying EARL1 and EARL2 compliant images, the second CNN identified 100% EARL1 compliant and 85% EARL2 compliant images correctly. The accuracy in the independent dataset was comparable to the cross-validation accuracy. The scan duration had almost no impact on the results.

**Conclusion:**

The two CNNs trained in this study can be used to retrospectively include images in a multi-center setting by, e.g., adding additional smoothing. This method is especially important for machine learning studies where the harmonization of images from different PET systems is essential.

**Supplementary Information:**

The online version contains supplementary material available at 10.1186/s40658-022-00468-w.

## Introduction

Positron Emission Tomography combined with Computed Tomography (PET/CT) using the tracer ^18^F-fluorodeoxyglucose (FDG) is nowadays widely used in oncology [1–3]. PET/CT has become part of the clinical routine for initial diagnosis, prognosis or treatment response assessment for cancer patients [4, 5].

To date, PET images are mainly inspected visually to, e.g., determine the tumor stage. In the last decade, the quantitative analysis of PET images has become more and more popular as it increases the reproducibility and objectivity of clinical decision making [6, 7].

For this purpose, PET images are routinely converted to Standardized Uptake Values (SUV) which normalizes the radioactivity displayed in the image by body weight and amount of injected tracer and thereby corrects for possible discrepancies across patients [8]. Next, quantitative assessment is performed by using basic SUV metrics, more complex image biomarkers such as radiomic features or most recently by deep learning analysis [9, 10]. Additional to SUV normalization also technical factors such as image reconstruction needs to be standardized in order to perform quantitative image analysis.

To perform reliable quantitative assessment, images need to be comparable across patients but also across institutions and PET/CT systems [11, 12]. However, due to the different image characteristics of scanner vendors or used reconstruction algorithms a wide variability in image quality across institutions and PET/CT systems remains which hampers a clinical implementation of quantitative image assessment [13]. To overcome these discrepancies, the European Association of Nuclear Medicine (EANM) provides certain guidelines and benchmark values to harmonize PET/CT images across centers [14, 15]. Two EANM Research Ltd. (EARL) standards exist and are established in participating institutions: one standard for older PET/CT systems harmonizing images with a lower resolution (EARL1) and the new EARL standard defined for modern PET cameras coming with higher resolution (EARL2) [16, 17]. Only images that are in accordance with these standards should be used for quantitative analysis.

To select harmonized reconstruction parameters, a scan of the NEMA image quality phantom is acquired and the reconstruction setting leading to activity recoveries in a certain range is chosen [15]. However, when including images retrospectively in a study, EARL accreditation might not have been in place or information in the DICOM header is missing such that reconstruction settings cannot be verified. Images that are not EARL compliant can be retrospectively converted to EARL compliant images by adding additional Gaussian smoothing [18] or data derived from the images by a data transformation method called COMBAT. To determine the amount of additional smoothing or the need for data transformation, it is of great interest to determine retrospectively if images that should be included in a study are meeting EARL requirements.

To the best of our knowledge, this is the first study that uses a convolutional neural network (CNN) to determine the EARL compliance of a PET image. For this purpose, we trained, validated, and tested two CNNs to determine if an image meets EARL standards. The first CNN is trained to determine if an image meets EARL standards in general (e.g., EARL1 or EARL2). The second CNN is then consecutively used to determine if an image that was identified as being EARL compliant is EARL1 or EARL2 compliant. Both CNNs were trained and cross-validated with data from three PET/CT systems from three PET scanner vendors (Siemens, Philips, and General Electrics) and externally validated on a forth PET/CT scanner in order to assess the generalizability of the trained algorithm.

## Materials and methods

### Datasets

#### Training and cross-validation

The dataset used for training and cross-validating the algorithm consists of 96 images from cancer patients acquired on three PET/CT systems: 36 images acquired on a Philips Gemini (Philips Medical Systems, Best, The Netherlands), 30 images on a Siemens Biograph mCT40 (Siemens, Knoxville, TN, USA), and 30 images on a General Electric Discovery system (General Electric, Boston, Massachusetts, USA). The training data included images from 17 lymphoma and 79 lung cancer patients. All images were reconstructed with (1) the locally clinically preferred, (2) EARL1 and (3) EARL2 compliant reconstruction settings and with a 120 s scan duration per bed position. The exact reconstruction settings for each scanner are displayed in Table 1. All data included in this study are taken from clinical routine. The use of the data was approved by the Institutional Medical Ethics Committees (case number VUMC 2018.029, UMCG 2017/489). All data were fully anonymized.

**Table 1:** Reconstruction settings for different scanner types

Scanner type	Clinical	EARL1	EARL2
Siemens biograph mCT40	PSF + 2 mm smoothing	OSEM + 6 mm smoothing	PSF + TOF + 5 mm smoothing
Philips Gemini	PSF + 2 mm smoothing	OSEM + 5 mm smoothing	PSF + TOF + 5 mm smoothing
GE discovery	PSF + TOF + 2 mm smoothing	PSF + TOF + 8 mm smoothing	PSF + TOF + 5 mm smoothing
Siemens Biograph Vision	PSF + TOF + 0 mm smoothing	PSF + TOF + 7 mm smoothing	PSF + TOF + 5 mm smoothing

### Independent testing datasets

To test the CNN performance, 30 images prospectively acquired on the Siemens Biograph mCT40 were used. These images were also reconstructed with clinical, EARL1, and EARL2 compliant reconstruction settings.

Moreover, 24 images acquired on a scanner that was not included in the training data (Siemens Biograph Vision) were used for independent external validation. This dataset included 9 lung cancer, 7 lymphoma, and 8 head and neck cancer patients. In order to determine if image noise has an impact on the CNN decision, all images acquired at the Biograph Vision were reconstructed with 30 s, 60 s, 120 s, and 180 s scan duration. The different scan durations were chosen to assess if the networks would also perform well when acquired in a hospital which scans their patients with other scan durations than 120 s per bed position.

### Training and validation of the CNN

Data preparation, as well as data analysis were performed with python 3.4. All implemented code used in this study can be found on Zenodo (https://doi.org/10.5281/zenodo.5540390).

### Data preparation and augmentation

**Fig. 1:**
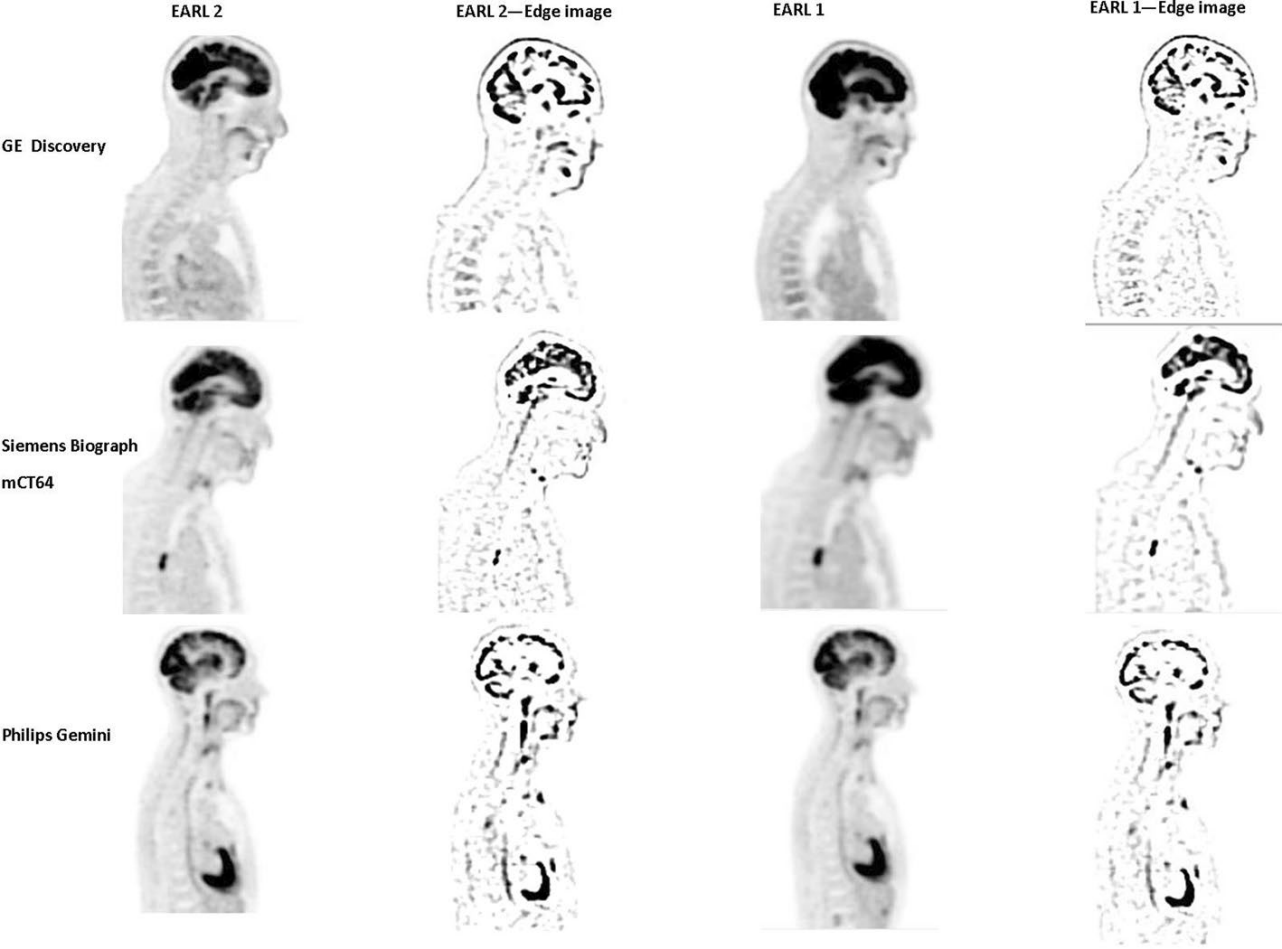
Original and edge enhanced image for EARL 1 and EARL 2 compliant images and the three PET systems included in the training dataset

First, all images were normalized to SUV units. Next, before images were used for training or validation, the images were converted to ‘edge images’. For this purpose, all images were blurred with a 6 mm Full-Width-At-Half-Maximum Gaussian kernel. Next, the blurred image was subtracted from the original image. The so generated ‘edge image’ pronounces the edges of high intensity areas while minimizing scanner specific noise. These ‘edge images’ are pronouncing the resolution of an image and resulted therefore in higher accuracies in training and validation than the original PET image (Additional file 1, Tables 1 and 2). An example is displayed in Fig. 1. The edge images were used for training, cross-validating, and independent validating the CNN.

**Table 2:** Training and cross-validation accuracy for the first CNN trained to separate clinical and EARL compliant reconstructions

Fold number	Training accuracy (%)	Validation accuracy for clinical reconstructions (%)	Validation accuracy for EARL compliant recons (%)
1	89	100	100
2	87	100	100
3	89	100	100
4	91	100	100
5	87	100	100

Before the conversion to edge images, all images were resampled to a cubic voxel size of 3 mm. After resampling, the images were cut or expanded to an image size of 300 × 200 pixels. Hereby, the images were padded with a constant value of 0 if their original image size was smaller than 300 × 200. If the image size was larger, they were cut by randomly choosing an image part with the required size. Hereby, the rows and columns for the cut were randomly assigned to lie between 0 and (300—original image height) or (200—original image width), respectively. This randomly cutting was performed to ensure that different body parts were present in the different images.

In order to avoid overfitting and to increase the number of training images, data augmentation was performed. Data augmentation included zooming the image, i.e., making it 10% larger or smaller, flipping the image either in vertical or horizontal direction, and adding a height or width shift of 10%.

### CNN architecture and training details

**Fig. 2:**
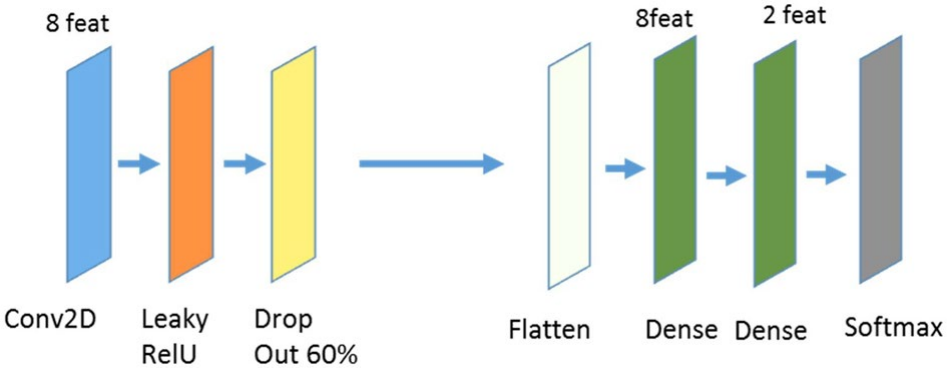
Workflow of the CNN used in this study: The convolutional block consists of a convolutional layer followed by a LeakyReLU layer with alpha set to 0.2. The convolutional layer consists of 8 filters. The dropout percentage is set to 0.6. The first dense layer consists of 8, the second one of 2 units

The CNNs were trained using the keras library version 2.2.4 with tensorflow backend. The trained CNNs as well as some example images and a manual how to train and validate the CNNs can be found on Zenodo. In this study, a 2D CNN is trained to classify single image slices (more details see below). The CNN architecture is displayed in Fig. 2. It consists of one convolutional block followed by a dropout layer and two dense layers. The convolutional block contains a convolutional layer with a kernel size of 3, a stride size of 2, and 8 filters. The initial weights of the convolutional layer are following a normal distribution. The dropout percentage in the dropout layer is set to 0.6 to avoid overfitting and to increase the generalizability of the CNN. The output of the dropout layer is flattened and fed into a dense layer with 8 units and a ReLU activation function. The second and last dense layer contains 2 units and a softmax activation function to perform the classification. The CNN is trained with 25 epochs and a batch size of 20. CNNs learn features that are important for the classification task automatically. To build a generalizable CNN, it is important to learn only features representing the specific task and not representing other details such as scanner specific noise. Therefore, it is essential to build a CNN that is generalizable and can be applied to data that is not included in the training process. In order to improve the generalizability, the CNN stopped training when the accuracy in the training set did not improve during 5 epochs. The CNN leading to the best validation accuracy in the training process is saved and used for further analysis.

To identify EARL compliant images, two CNNs with the described architecture were trained. Two separate CNNs were trained as the use of only one CNN with three outcomes (Clinical, EARL1, and EARL2) resulted in worse performance (see Additional file 1, Table 3). The first CNN was trained to separate images that are meeting EARL standards (EARL1 and EARL2) from images that are not EARL compliant. For training this CNN, the clinical reconstructions of the three scanners as well as the EARL2 compliant images from the GE and the Siemens system and the EARL1 compliant images from the Philips system were used. This subselection was performed to avoid data imbalance. The second CNN was trained to determine if an image that was identified to be EARL compliant is compliant with either EARL1 or EARL2 standards. For this task, EARL1 and EARL2 compliant images of all scanners were used for training.

**Table 3:** Training and cross-validation accuracy for the second CNN trained to classify EARL1 and EARL2 compliant reconstructions

Fold number	Training accuracy (%)	Validation accuracy for EARL1 compliant reconstructions (%)	Validation accuracy for EARL2 compliant recons (%)
1	88	100	83
2	89	100	87
3	87	100	87
4	91	100	78
5	93	100	87

### Slice selection

In the present study, only sagittal slices were used for classification as they showed the best results in initial experiments as compared to using axial or coronal slices (data not shown). For the first CNN trained to separate clinical and EARL compliant images, ten slices in the middle of the patient were chosen. Hereby, the middle of the image was determined and the five slices on the left-hand and the five slices on the right-hand side were included in the training dataset. For the second CNN trained to separate EARL1 and EARL2 compliant images, slices with a high gradient (i.e., with a high uptake) were chosen. For this purpose, from the forty slices in the middle of the patient, the ten slices with the highest edge intensity values were selected. In both cases, the mean probability value of the ten slices was calculated and used for final classification.

As described above, images were randomly cut before they were used as input to the network. To assess if the random cut had an impact on the CNN results, ten randomly selected images were analyzed using five different random cuts. The mean, standard deviation, and coefficient of variation (COV, defined as ratio of standard deviation and mean value) of these ten CNN probabilities were calculated and compared.

### Model performance

For training and cross-validating the CNNs, the training dataset was split in five parts such that each part contained a comparable number of images from each scanner. Fivefold cross-validation was performed so that each part served once as validation dataset. The performance of the trained CNN was evaluated by analyzing prediction accuracy. As the softmax layer of the CNN gives certainty about its decision in terms of a probability (1 for a completely certain decision, 0.5 for an uncertain decision), the mean probability of the ten image slices was used for final classification. For the first CNN, all images with a probability equal or above 0.5 to be clinical/EARL compliant images were considered to be clinical/EARL compliant images. While for the second CNN, images with a corresponding mean probability of 0.4 or higher were considered as being EARL2 compliant while images with a mean probability of 0.6 or higher were considered as EARL1 compliant.

Last, the CNNs were trained on the whole training dataset and then applied to the independent validation datasets.

## Results

### Cross-validation accuracy

**Fig. 3:**
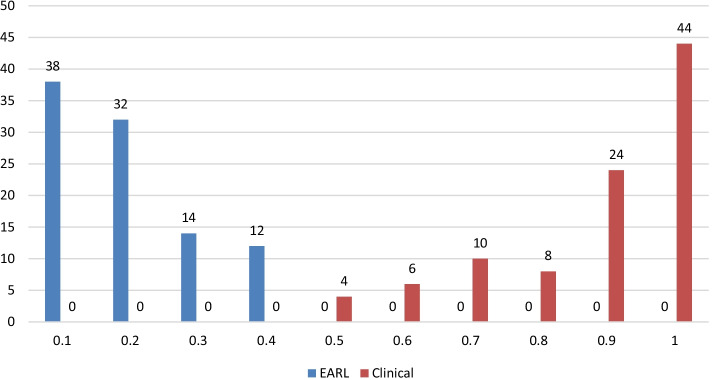
Probabilities of the first network to identify images reconstructed with clinical reconstruction settings. If the probability is below 0.5 for an EARL compliant image, this image is correctly classified As illustrated, the network can separate well between EARL compliant and clinical reconstructions

The training and cross-validation accuracies of the first CNN trained to separate EARL compliant and clinical reconstructions are listed in Table 2. Note that the training accuracy represents the accuracy for single slices, while the cross-validation accuracy is calculated image per image by using the mean probability of ten patient slices (as explained above). For all folds, all clinical and all EARL compliant reconstructions were correctly identified. The probabilities of the CNN representing the confidence of its decision that an image is a clinical reconstruction are displayed in Fig. 3.

**Fig. 4:**
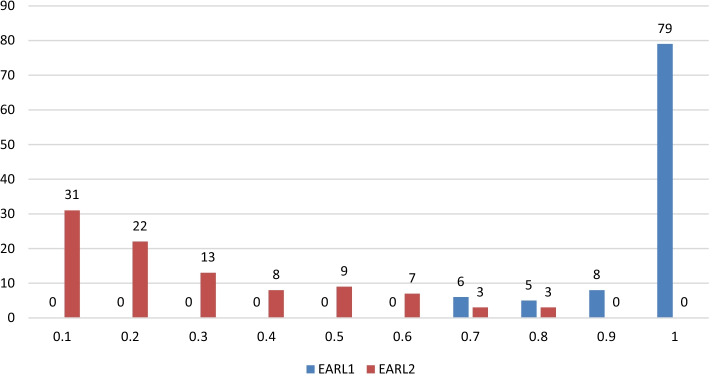
Probabilities of the second network. These probabilities are representing the certainity of the network that an image is EARL1 compliant. If the probability is below 0.6 for an EARL2 compliant image, the image is still correctly classified. The network can identify EARL1 reconstructions very well why it has more difficulties with identifying EARL2 compliant images. As can be seen, by setting the threshold to separate these two reconstructions to 0.6 the overall accuracy increases

The accuracy values for the second CNN trained to separate EARL1 and EARL2 compliant images are listed in Table 3. EARL1 compliant images were always correctly identified. In contrast, in each fold, some EARL2 compliant image were incorrectly classified as being EARL1 compliant. The majority of the misclassified images were patients with small tumor lesions and corresponding edge images with low gradient values. The probabilities of the CNN decision that the images are EARL1 compliant are displayed in Fig. 4. This Figure also illustrates the chosen threshold of 0.6.

### Accuracy independent validation dataset

In the independent testing dataset, all images were correctly classified by the first CNN. The second CNN trained to separate EARL1 and EARL2 compliant images identified all EARL1 compliant images correctly while 18% of the EARL2 compliant images were incorrectly classified. As in the cross-validation datasets, the incorrectly classified images were patients with low tumor load and therefore low edge values (Table 4).

**Table 4:** Accuracy of the first CNN applied to data from the Siemens Biograph Vision which was not included in the training data

	Clinical (%)	EARL1 (%)	EARL2 (%)
Correctly identified images—180 s	84	100	100
120 s	100	100	100
60 s	100	100	100
30 s	100	100	96

The accuracy of the first CNN when applied to the data acquired on the Siemens Biograph Vision are listed in Table 4 for 30 s, 60 s, 120 s, and 180 s scan duration. For the same scan duration as the training data (120 s), all reconstruction settings were correctly identified.

The scan duration had only for three clinical images acquired at the Biograph Vision with scan duration 180 s an impact on the CNN prediction. The images were incorrectly identified as being EARL compliant by the first CNN, the second CNN identified them to be EARL2 compliant. Here, the probability given by the CNN was for all scan durations around 50% while it dropped slightly below 50% for 180 s scan duration. Also one EARL2 compliant image reconstructed with 30 s scan duration was incorrectly classified as clinical reconstructions while the same scan reconstructed with longer scan durations was correctly classified. For all other images, the scan duration had no impact on the binary decision. However, the CNN probability and therefore the certainty of the CNN dropped around 10% for images with scan durations different from 120 s.

Table 5 contains the accuracy of the CNN trained to separate EARL1 and EARL2 compliant images for different scan durations. For this CNN, the scan duration had no impact on the results. All images were correctly identified for all scan durations.

**Table 5:** Accuracy of the second CNN applied to images reconstructed with different scan durations acquired on the Biograph Vision

	EARL1 (%)	EARL2 (%)
Correctly identified images—180 s	100	100
120 s	100	100
60 s	100	100
30 s	100	100

### Impact of random cut on CNN results

Table 6 contains the mean, standard deviation, and COV of the CNN probabilities when the images were cut five times randomly. The standard deviation was small (range from 0.0051 to 0.098) and the COV yielded values between 0.0016 to 0.11. Both results illustrate that the random cut of the image has almost no impact on the CNN results.

**Table 6:** Mean, standard deviation, and coefficient of variation of CNN probabilities for five different random cuts. For each image, the standard deviation is small. Therefore, the CNN results are independent of the random cut

	Mean	Std. dev	Coefficient of variation
Image 1	0.89	0.098	0.11
Image 2	0.98	0.049	0.05
Image 3	0.78	0.0078	0.01
Image 4	0.68	0.0051	0.0075
Image 5	0.82	0.013	0.0016
Image 6	0.94	0.0041	0.0043
Image 7	0.57	0.0032	0,0056
Image 8	0.99	0.087	0.088
Image 9	0.81	0.0059	0.0072
Image 10	0.97	0.042	0.043

## Discussion

In this study, we were the first to train, cross-validate, and independently validate an automatic image quality control for PET images. As such the trained CNNs could be used in clinical multi-center studies to determine if images from different institutions are in compliance with the EARL standards and can be used together. To make the methods and results publicly available, a python script that takes one image or a series of nifty images as input and displays the corresponding reconstruction setting is available on Zenodo and can be used by the community.

Quality control for PET images is an important task. With the increasing use of machine and deep learning in nuclear medicine, where the amount of training data is limited, it is especially important to use images yielding similar image quality. To date, image quality in PET images can be assessed by drawing manually a sphere in the patient liver and comparing the mean liver intensity value across patients. However, the mean liver value gives mainly information about the correct SUV and does not necessarily indicate if the used reconstruction settings are comparable [12].

While details about the used reconstruction setting are listed in the DICOM header, only very experienced users can identify if the reconstruction setting is compliant with EARL standards. Additionally, due to anonymization or conversion to other image formats such as, e.g., nifti this information is often incomplete.

In this study, the two dimensional image slices were used as input to the CNN and not—as for other tasks- the 3D information of the whole image. By using 2D slices, the number of training data was enlarged, thereby possibly increasing the classification performance. Moreover, in contrast to segmentation or other tasks, the 3D information of the image is not important as only the noise structure and resolution of the image is important for the classification.

Several strategies were used in this study to guarantee the generalizability of the CNN and to avoid overfitting. As PET images come with a low spatial resolution and less image details than, e.g., CT images, a lower number of features and CNN layers might be an advantage [7]. To build generalizable CNNs is an important challenge as CNNs contain normally a large number of learnable features while the amount of training data is often limited. Therefore, the CNN used in this study is very sparse, containing only a few number of layers and learnable features. A large dropout percentage was chosen in order to avoid overfitting. The comparable performance of training, cross-validation, and independent external testing underlines the generalizability of the proposed CNNs. The low amount of layers and features seems appropriate because of the lack of details that are needed to identify the image resolution (i.e., EARL1, EARL2 or clinical image quality). In our experience, when using more features and layers the CNNs learned scanner specific details and were not able to generalize to unseen data.

Some EARL2 compliant images were incorrectly identified as being EARL1 compliant. This was especially the case for patients with low tumor load. In these cases, the edge images contained only few edges what might be the reason for the misclassification. However, as the CNN made the right decision in the majority of the cases, it may still be possible to assess if an imaging site complies with an EARL standard by looking at the overall classification across all images from an imaging site.

The scan duration had in our study only a low impact on the results. However, to make the CNN more stable and to achieve that the scan duration has no impact at all, it might be necessary to train the network with images acquired with different scan durations per bed position. This would increase the stability and generalizability of the proposed method.

Even though the images used for training contained mainly lung cancer images, the network performed well on other cancer types such as lymphoma or head and neck cancer in the independent validation dataset. This result indicates that the network can perform well for various cancer types even though these cancer types were not present in the training dataset. These results need to be confirmed in a larger dataset. The present study as well as the EARL accreditation focuses on oncological PET images. However, also for neurological images, harmonized image reconstructions are essential in order to compare images across institutions [19]. Therefore, future studies could focus on the use of CNNs to determine image quality for neurological PET images.

The present study has some limitations. First, the network misclassified images yielding no or only small (smaller than 5 mL) tumor lesions with a maximum uptake below 3 SUV. However, by knowing this limitation, the user can focus on identifying the right reconstruction setting using images with larger tumor lesions. Second, the network was trained with a small number of images what could lead to a low generalizability of the trained network. By including an external validation dataset from a scanner not included in the training data, we tried to assess the generalizability of the CNN and achieved comparable results to the training data. Still, this does not guarantee that the network will perform well for other scanners. In general, to improve the stability of the proposed method, retraining the network with data coming from more scanners would be of advantage. Especially for newer generations of PET scanners it might be necessary to retrain the model with data from these scanners in order to achieve good results. Additionally, the network was only trained with images acquired with 120 s scan duration. If in the future, a lower scan duration will be feasible for clinical studies, it will likely also be of advantage to include images with other scan durations in the training process. At the moment, one GPU is necessary to train and retrain the network. However, by reducing the image size, it will be possible to train the network also on a CPU what would make the method also feasible for institutions without access to computer clusters. Therefore, we made the trained networks as well as the code used in this paper publicly available. Users can retrain the network with data from other scanners such that the generalizability of the proposed method can be further improved.

## Conclusion

We trained, cross-validated and independently validated two consecutive CNNs that can automatically identify EARL compliant images. Moreover, the CNNs are able to separate if an image is meeting older or newer EARL standards. By using the proposed CNNs, images that are not EARL compliant can be adjusted retrospectively (by adding additional smoothing) so that they become EARL compliant, thus giving the opportunity to retrospectively include images in a multi-center setting. This method can be used in multicenter studies where the harmonization of images from different PET systems is essential.

## Supplementary Information

**Additional file 1. Supplemental Table 1**. Accuracy for CNN trained to identify clinical and EARL compliant reconstructions when using original SUV images.** Supplemental Table 2**. Accuracy for CNN trained to identify EARL1 and EARL2 compliant reconstructions when using original SUV images.** Supplemental Table 3**. Accuracy for CNN when trained with three outcomes (Clinical, EARL1, EARL2).

## Data Availability

The datasets used and/or analyzed during the current study are available from the corresponding author on reasonable request.
