# Supplementary material for: Convolutional neural networks for automatic image quality control and EARL compliance of PET images
Source: EJNMMI Phys. 2022 Aug 9;9:53. doi: 10.1186/s40658-022-00468-w (PMC9363539; doi:10.1186/s40658-022-00468-w)
Supplement: Supplementary file 1 — Additional file 1. Supplemental Table 1. Accuracy for CNN trained to identify clinical and EARL compliant reconstructions when using original SUV images. Supplemental Table 2. Accuracy for CNN trained to identify EARL1 and EARL2 compliant reconstructions when using original SUV images. Supplemental Table 3. Accuracy for CNN when trained with three outcomes (Clinical, EARL1, EARL2). [file 40658_2022_468_MOESM1_ESM.docx]

| Fold number | Training accuracy | Validation accuracy for clinical reconstructions | Validation accuracy for EARL compliant recons |
| --- | --- | --- | --- |
| 1 | 71% | 50% | 100% |
| 2 | 75% | 5% | 100% |
| 3 | 69% | 30% | 100% |
| 4 | 65% | 10% | 100% |
| 5 | 76% | 41% | 100% |

Supplemental Material

1. Accuracy when using original images for training/cross-validation

Supplemental Table 1: Accuracy for CNN trained to identify clinical and EARL compliant reconstructions when using original SUV images

| Fold number | Training accuracy | Validation accuracy for EARL2 reconstructions | Validation accuracy for EARL1 recons |
| --- | --- | --- | --- |
| 1 | 75% | 30% | 100% |
| 2 | 78% | 30% | 100% |
| 3 | 81% | 30% | 100% |
| 4 | 71% | 30% | 100% |
| 5 | 76% | 30% | 100% |

Supplemental Table 2: Accuracy for CNN trained to identify EARL1 and EARL2 compliant reconstructions when using original SUV images

1. Accuracy when using one CNN with three different outcomes (clinical, EARL2, and EARL1)

| Fold number | Training accuracy | Validation accuracy for clinical reconstructions | Validation accuracy for EARL2 compliant recons | Validation accuracy for EARL1 compliant recons |
| --- | --- | --- | --- | --- |
| 1 | 72% | 100% | 54% | 71% |
| 2 | 71% | 93% | 49% | 68% |
| 3 | 74% | 87% | 57% | 73% |
| 4 | 69% | 87% | 41% | 71% |
| 5 | 78% | 93% | 59% | 76% |

Supplemental Table 3: Accuracy for CNN when trained with three outcomes (Clinical, EARL1, EARL2)
